# Identification and Characterization of *Bacillus tequilensis* GYUN-300: An Antagonistic Bacterium Against Red Pepper Anthracnose Caused by *Colletotrichum acutatum* in Korea

**DOI:** 10.3389/fmicb.2022.826827

**Published:** 2022-03-02

**Authors:** Hyeok-Tae Kwon, Younmi Lee, Jungyeon Kim, Kotnala Balaraju, Heung Tae Kim, Yongho Jeon

**Affiliations:** ^1^Department of Plant Medicals, Andong National University, Andong, South Korea; ^2^Agricultural Science and Technology Research Institute, Andong National University, Andong, South Korea; ^3^Department of Plant Medicine, Chungbuk National University, Cheongju, South Korea

**Keywords:** *Bacillus tequilensis*, anthracnose, fungicide resistance, fungal pathogen, genome sequence, microbial pesticide

## Abstract

Anthracnose is a fungal disease caused by *Colletotrichum* species and has detrimental effects on many crops, including red pepper. This study used *Bacillus tequilensis* GYUN-300 (GYUN-300), which exhibit antagonistic activity against the fungal pathogen, *Colletotrichum acutatum*. This pathogen causes anthracnose that manifests primarily as a fruit rot in red pepper. There have been little efforts to identify antagonistic bacteria from mushrooms; this strain of bacteria was identified as *B. tequilensis* using BIOLOG and 16S rDNA sequencing analysis. The genetic mechanism underpinning the biocontrol traits of GYUN-300 was characterized using the complete genome sequence of GYUN-300, which was closely compared to related strains. GYUN-300 inhibited mycelial growth and spore germination of *C. acutatum* under *in vitro* conditions. Important antagonistic traits, such as siderophore production, solubilization of insoluble phosphate, and production of lytic enzymes (cellulase, protease, and amylase), were observed in GYUN-300, These trains promoted growth in terms of seed germination and vigorous seedling growth compared to the non-treated control. When red pepper fruits were treated with GYUN-300, the preventive and curative effects were 66.6 and 38.3% effective, respectively, in wounded red pepper fruits; there was no difference between the preventive and curative effects in non-wounded red pepper fruits. Furthermore, GYUN-300 was resistant to several commercial fungicides, indicating that GYUN-300 bacterial cells may also be used synergistically with chemical fungicides to increase biocontrol efficiency. Based on *in vitro* results, GYUN-300 played a role to control anthracnose disease effectively in field conditions when compared to other treatments and non-treated controls. The results from this study provide a better understanding of the GYUN-300 strain as an effective biocontrol agent against red pepper anthracnose; this form of biocontrol provides an environment-friendly alternative to chemical fungicides.

## Introduction

Red pepper (*Capsicum annuum* L.) is a vegetable crop belonging to the Solanaceae family, with the cultivation area of 33,373 hectares. The total production of chili pepper recorded as 92,756 tons in the year 2021 in Korea^1^ (Kim et al., 2014). To meet the demands of a growing population, an increased production of red pepper along with other vegetables are required in Korea. Therefore, a large quantity of chemical pesticides is utilized annually to control various bacterial, fungal, and viral diseases on red pepper crops in Korea (Hong et al., 2015). Among all pathogens that cause diseases in red peppers, anthracnose caused by a wide range of *Colletotrichum* species results in serious losses of fruits during the pre- and post-harvest stages (Ali et al., 2016), while occasionally damaging the stem and foliage (Mishra et al., 2018; Mongkolporn and Taylor, 2018). To date, 24 *Colletotrichum* species infecting red peppers have been identified; the most common species are *Colletotrichum acutatum* and *Colletotrichum gloeosporioides*, which inflict serious damage to red pepper (Kim et al., 2008; Parisi et al., 2020). The fungal species of *Colletotrichum* cause anthracnose in several plants, including red peppers (Siddiqui and Ali, 2014). Anthracnose was responsible for a 10% annual reduction in pepper productivity in South Korea (Kim et al., 2008). Anthracnose in red pepper is associated with several *Colletotrichum* species, including *C. acutatum* (Harp et al., 2014), this may also infect other fruit and vegetable crops (Han et al., 2015). Red pepper anthracnose is typically characterized by dark brown to black, circular water-soaked spots with concentric rings of black acervuli developing beneath the skin of the fruit (Parisi et al., 2020); the spots are often numerous and coalesce, causing softening and fruit rot (Montri et al., 2009).

Anthracnose is usually controlled by the application of chemical fungicides in field conditions. The increased use of chemical fungicides has led to the development of resistance in fungal strains (Onyeka et al., 2006). In particular, *C. acutatum* is resistant to specific chemical fungicides, including carbendazim, benomyl, and copper oxide (Peres et al., 2002; Jayasinghe and Fernando, 2009). The continuous use of chemical pesticides increases public concern regarding the risks associated with hazardous residues on agricultural products and its adverse effects on biodiversity in ecosystems (Meena et al., 2020). In addition, disease control by chemical pesticides is cost-effective for farmers in developing countries (Wesseling et al., 1997; Bordoh et al., 2020). For these reasons, biological control using antagonistic microorganisms has emerged as environment-friendly alternative to control plant diseases (Köhl et al., 2019; De la Lastra et al., 2021). Antagonistic microorganisms produce a variety of secondary metabolites to combat plant pathogens (Hossain et al., 2015; Köhl et al., 2019; Cellini et al., 2021). They induce resistance against pathogen infections in plant tissues without direct antagonistic interactions with the pathogen itself (Sharma et al., 2019). Similarly, a previous study by Ali et al. (2015) reported that the red pepper anthracnose was suppressed by an antimicrobial organic compound, propolis under post-harvest conditions. Additional indirect interaction with pathogens occurs during competition for nutrients and space, antibiotic production, colonization, induced systemic resistance (ISR), and parasitism against target plant pathogens (Kloepper et al., 2004; Kim et al., 2012); however, antagonistic microorganisms often do not exhibit consistent disease suppression compared to the commercial fungicides (Sang et al., 2013). Plant growth-promoting rhizobacteria (PGPR) are antagonistic to plant pathogens as they produce secondary metabolites, including siderophores, lytic enzymes, and antibiotics (Conn et al., 2008); they also produce plant growth regulators, such as indole-3-acetic acid (IAA), and solubilization of insoluble phosphates (Goswami et al., 2016).

There are several studies on antibiotic production by antagonistic microbes, such as *Bacillus* sp., *Pseudomonas* sp., and *Streptomyces* sp. (Beneduzi et al., 2012; Ngalimat et al., 2021; Yang et al., 2021). *Bacillus* spp., produce spores, which may be used to develop an effective microbial biopesticide formulation in the form of a biocontrol agent (BCA). Previously, many studies have demonstrated that several secondary metabolites produced by antagonistic bacteria play key roles in controlling various phytopathogens (Han et al., 2015). To date, there have been no reports on the isolation of these bacteria from the edible mushroom, locally known as “Sanghwang.” It is a popular medicinal mushroom wildly cultivated in China, Japan, and Korea (Chen et al., 2016). This is grown on different trees, such as Oak and mulberry (Sliva, 2010), and being used as a traditional medicine to treat various health disorders, such as inflammation, gastroenteric, lymphatic diseases, and cancers (Jang et al., 2015). This further may exhibits biocontrol potential against anthracnose caused by *C. acutatum* in red pepper. This study sought to meet two key objectives: (i) evaluate the potential efficacy of the selected PGPR strain, *Bacillus tequilensis* GYUN-300, as a BCA against red pepper anthracnose under *in vitro* and field conditions; and (ii) evaluate its complete genome sequence for determining divergent genomic characteristics among other *Bacillus* strains.

## Materials and Methods

### Isolation of Antagonistic Bacteria From Mushroom

Fresh sanghwang mushrooms were purchased from the local traditional market and thoroughly washed with water in the laboratory. Thereafter, 5 g of mushroom was ground in 10 mL sterilized distilled water (SDW) and diluted to 10^–8^. Next, 100 μL of the diluted samples were plated onto nutrient agar plates; single colonies were selected 48 h after incubation at 28°C. Of the 20 total isolates obtained, only one isolate (i.e., GYUN-300) was selected for further study based on primary screening on *in vitro* antagonistic activity.

### Source of Fungal Pathogens Used

The pathogen *C. acutatum* KACC42403 as obtained from Korean Agricultural Culture Collection (KACC), Agricultural Microbiology Division, RDA, South Korea. Other two pathogens, *C. acutatum* ANBPC, and *C. acutatum* ANYPC, were isolated from the infected fruits. After cutting the small sections of the lesion from the infected fruit, they were surfaces sterilized in 70% ethanol for 30 s and were rinsed twice in SDW. Thus, surface sterilized tissues were placed onto potato dextrose agar (PDA) medium. The fungal colonies were obtained after incubating the plates at 25°C for 7 days. The isolates ANBPC and ANYPC were recovered from these colonies. The cultures were stored on PDA plates at 10°C for further use.

### *In vitro* Antagonistic Activity of GYUN-300

Bacteria isolated from sanghwang mushrooms were diluted to 10^–6^ and 10^–5^ in SDW and placed onto tryptic soy agar (TSA) plates. Out of several bacterial isolates, only GYUN-300 was selected based on its *in vitro* antagonistic activity against *C. acutatum* isolates. The isolate GYUN-300 was maintained at −80°C in tryptic soy broth (TSB) with glycerol (20%) for long-term storage. To prepare bacterial suspensions, a culture from the −80°C stock was grown on TSA plates for 3 days at 28°C; single colonies were transferred to TSB and incubated for 48 h at 28°C under shaking conditions (150 rpm). Bacteria were pelleted after centrifugation for 5 min at 8,000 × *g* and suspended in SDW to obtain a final concentration of 1 × 10^6^ colony-forming units (CFU)/mL prior to application. The *in vitro* antifungal activity of bacterial cell suspensions of GYUN-300 was tested against *C. acutatum* isolates using a dual culture plate assay. Briefly, the fungal pathogenic isolates, *C. acutatum* KACC42403, *C. acutatum* ANBPC, and *C. acutatum* ANYPC were cultured on potato dextrose agar (PDA) medium for 7 days at 25°C to obtain mycelial plugs for the *in vitro* antagonism tests. The mycelial plugs (6 mm in diameter) of *C. acutatum* were placed onto the center of the PDA plates, and 10 μl of antagonistic bacterial GYUN-300 cell suspensions (1 × 10^6^ CFU/mL) impregnated on sterile paper discs (6 mm in diameter) were placed on the top, bottom, right, and left sides of the plate 30 mm away from the center of the plate; plates treated with SDW served as the non-treated control. The growth inhibition distance between the bacterial suspension and pathogen was measured 7 days after incubation at 25°C in comparison with the non-treated control. Each treatment consisted of five replicates (Petri dishes), and the experiment was carried out at least twice.

### Preparation of Fungal Pathogen Inocula

Conidial suspensions (100 μL) were spread onto PDA plates and incubated at 25°C for 7 days. The conidia were harvested by pouring SDW onto a PDA plate containing pathogenic fungi, and scraped. The suspensions were filtered through a double-layered cheesecloth. The concentration of spore suspensions was adjusted to 10^5^ conidia/mL using a hemocytometer prior to its application.

### Inhibition of Spore Germination of *Colletotrichum acutatum* Through Treatment With GYUN-300 Bacterial Cell Suspensions

Conidial germination and appressorium formation from *C. acutatum* were tested on a cover glass surface treated with GYUN-300 bacterial suspensions and its culture filtrate (CF) using a method described by Kim et al. (1998). Briefly, conidia from the cultures grown on PDA plates for 7 days were harvested and washed twice with ice-cold SDW. Conidial suspensions (10^6^ CFU/mL) of 100 μL were mixed into Eppendorf tubes containing bacterial cell suspensions of GYUN-300 or its CF, and incubated for 48 h at 25°C. Conidial germination and the formation of appressorium and primary hyphae in the GYUN-300 or its CF treatment were assessed at different durations (i.e., 8, 16, 24, 32, 40, and 48 h) in Petri dishes containing moist paper. At minimum, 50 measurements were carried out per structure with a ProgRes SpeedXT *^core^* 3 Imager microscope using a differential interference contrast illumination.

### Inhibition of Fungal Mycelial Growth by Volatile Compounds From GYUN-300

An I-plate assay (Sharifi and Ryu, 2016) and dual culture plate assays (Chaurasia et al., 2004) were carried out to evaluate the ability of the GYUN-300 stain to inhibit fungal growth through the production of volatile compounds. For the I-plate assay, one compartment of the I-plate contained TSA medium to inoculate GYUN-300, while the other side contained PDA to culture the fungal pathogen, *C. acutatum* KACC42403; the plate treated with SDW served as the non-treated control. Mycelial growth inhibition was observed 7 days after incubation at 25°C. For the dual culture plate assay, a Petri dish containing PDA medium was inoculated with a pathogenic mycelial plug on one side just 10 mm from the edge of the plate, and cell suspensions of GYUN-300 were used to inoculate the other side just 10 mm from the edge of the plate, by streaking on the same Petri dish without contacting the fungal pathogen. The inhibitory effect of GYUN-300 on fungal mycelia was measured 7 days after incubation at 25°C. Each test was conducted in triplicate, and three independent measurements were taken for each plate.

### Identification of Antagonistic Bacteria Using MicroLog System

The putative *Bacillus* isolate was tested for the utilization of 95 carbon sources using the BIOLOG program (Jeon et al., 2003). Briefly, bacterial cells cultured on TSA medium for 24 h at 28°C were suspended in an inoculating fluid (i.e., 0.4% NaCl, 0.03% Pluronic F-68, and 0.01% gellan gum). They were inoculated onto GENIII MicroPlates (BIOLOG Inc., BiOLOG GP MicroPlateTM, Hayward, CA, United States), and incubated at 28°C. After 24 h of incubation, turbidity in the wells was measured using a MicroLogTM 3-Automated Microstation system (BIOLOG, Hayward, CA, United States). Bacteria were identified using the MicroLog Gram-positive database (ver. 4.0; BIOLOG).

### Molecular Identification of GYUN-300

The selective isolate, GYUN-300, was subjected to molecular identification based on the sequence homology of its 16S rDNA gene (Weisburg et al., 1991). The genomic DNA (gDNA) of GYUN-300 was isolated using a kit (BioFact Genomic DNA Extraction Kit, Biofact Co., Seoul, South Korea) as per the manufacturer’s instructions. The 16S rDNA gene was amplified using polymerase chain reaction (PCR) with Taq DNA polymerase, and primers 27F (5’-AGA GTT TGA TCM TGG CTC AG-3’) and 1492R (5’-GGY TAC CTT GTT ACG ACT T-3’) were used for amplification. The thermal cycling conditions were: denaturation at 94°C for 5 min followed by 35 cycles at 94°C for 30 s, annealing at 55°C for 30 s, and extension at 72°C for 1 min. At the end of the cycle, the reaction mixture was held at 72°C for 10 min, and then cooled to 4°C. The PCR product was purified using a PCR gel purification kit (BIOFACT Co., Seoul, South Korea), according to the manufacturer’s instructions. The purified PCR product was sequenced using an automated sequencer (Genetic Analyzer 3130; Applied Biosystems, Carlsbad, CA, United States), in which the same primers were used utilized. The sequence was compared with the reference bacterial species contained in a genomic database using the Basic Local Alignment Search Tool (BLAST) from the National Center for Biotechnology Information (NCBI). Sequence alignment and phylogenetic tree construction were carried out using the MEGA 4.0 program (Biodesign Institute, Tempe, AZ, United States).

### GYUN-300 Whole-Genome Sequence Analysis and Annotation

Bacterial gDNA was extracted using a FastDNA™ SPIN Kit for Soil (MP Biomedicals, Santa Anna, CA, United States), according to the manufacturer’s instructions. The concentration of extracted DNA was determined using a Qubit 2.0 fluorometer (Invitrogen, Carlsbad, CA, United States). The contamination of DNA or the cultured strain was tested by sequencing the 16S rRNA gene using an ABI 3730 DNA sequencing machine (Applied Biosystems, Foster City, CA, United States). The integrity of the gDNA was verified using agarose gel electrophoresis, and quantified using a Qubit 2.0 fluorometer (Invitrogen, Carlsbad, CA, United States). Then, sequencing libraries were prepared according to the manufacturer’s instructions for 20 kb template preparation using the BluePippin Size-Selection System using PacBio DNA Template Prep Kit 1.0. Briefly, 10 μg of the gDNA was sheared to 20 kb using g-tubes (Covaris); they were then purified, end-repaired, and the blunt-end SMARTbell adapters were ligated. The libraries were quantified using a Qubit 2.0 fluorometer (Invitrogen, Carlsbad, CA, United States) and qualified using the DNA 12000 chip (Agilent Technologies, Waldbronn, Germany). Subsequently, the libraries were sequenced using PacBio P6C4 chemistry in the 8-well SMART Cell v3 in PacBio RSII.

The genome of the GYUN-300 strain was constructed *de novo* using PacBio sequencing data; sequencing analysis was carried out at Chunlab, Inc., PacBio sequencing data were assembled with PacBio SMRT Analysis 2.3.0, using the HGAP2 protocol (Pacific Biosciences, Menlo Park, CA, Inc., United States); the resultant contigs of the PacBio sequencing data were prototyped using Circlator 1.4.0 (Sanger Institute). Gene-finding and functional annotation pipeline of the whole-genome assembly used in the EzBioCloud genome database, and the protein-coding sequences (CDSs) were predicted using Prodigal 2.6.2 (Hyatt et al., 2010). The gene encoding tRNA was identified using the tRNAscan-SE 1.3.1 (Schattner et al., 2005), while the rRNA and other non-coding RNAs were analyzed using the Rfam 12.0 database (Nawrocki and Eddy, 2013). The CRISPRs were detected by PilerCR 1.06 (Edgar, 2007) and CRT 1.2 (Bland et al., 2007). The CDSs were classified into groups based on their roles, referencing the orthologous groups^2^ (EggNOG 4.5) (Powell et al., 2014). For more functional annotation, the predicted CDSs were compared with the Swissprot (UniProt Consortium, 2015), KEGG (Kanehisa et al., 2014), and SEED (Overbeek et al., 2005) databases using the UBLAST program (Edgar, 2007).

### *In vitro* Enzyme Activity by Antagonistic Bacteria

The ability of bacteria to produce various enzymes, such as cellulase, protease, and amylase was tested under *in vitro* conditions. Cellulase production was assessed by inoculation of GYUN-300 cell suspensions on TSA media supplemented with 5 g/L carboxymethyl cellulose (CMC). Sterilized filter paper discs (6 mm diameter) impregnated with 10 μL bacterial cell suspensions were placed on Petri dishes at four sides 15–20 mm away from the edge of plates, then incubated at 28°C for 5 days. After pouring 0.1% Congo Red solution onto the surface of Petri dishes, and retained for 5–10 min; Plates were then de-stained using a 1 M NaCl solution. The development of a clear halo around the colonies indicated a positive reaction and the production of cellulase by bacteria. For protease activity, bacterial isolates were inoculated on gelatin media (i.e., gelatin 5 g, beef extract 3 g, protease peptone 5 g, agar 15 g, distilled water 1,000 mL); plates were then incubated at 28°C for 2–3 days. After dispensing plates with 1% tannic solution for 5 min for staining, they were washed with SDW; Protease activity was recorded through the development of a clear zone (halo) around the colonies, indicating that proteins were hydrolyzed by the bacteria. Amylase production was assessed through the inoculation of the GYUN-300 isolate on TSA media supplemented with 0.5% soluble starch. Sterile paper discs (6 mm) impregnated with bacterial cell suspensions were plated on solid media, and Petri dishes were incubated at 28°C for 2–3 days. After pouring iodine solution (0.3 g iodine and 0.6 g KI/L) on the surface of Petri plates, any clear halo development around colonies was indicative of a positive reaction and the production of amylase and degradation of starch by bacteria.

### Determination of Siderophore Production and Phosphate Solubilization

The procedure developed by Schwyn and Neilands (1987) using Chrome Azurol S (CAS) media was used to evaluate the ability of bacteria to produce siderophores. Briefly, 10 μL GYUN-300 bacterial suspensions were inoculated on iron-free King B solid media and incubated at 28^°^C for 2–3 days. Then, CAS agar (15 mL) was poured onto the GYUN-300 bacterial cultures, and after a few hours the plates were checked for the appearance of a halo around bacterial colonies, with a color change from blue to orange. The halo diameter was calculated by subtracting the diameter of the colony from the total diameter of the halo and the colony, a procedure developed by Gaur (1990) was used to determine the ability of bacteria to degrade the inorganic phosphates from the soil. Briefly, sterile paper discs (6 mm diameter) impregnated with 10 μL of GYUN-300 bacterial suspensions were placed onto the surface of tricalcium phosphate medium (i.e., glucose 10 g, tricalcium phosphate 5 g, MgCl_2_ 6H_2_O 5 g, MgSO_4_ 7H_2_ 0.025 g, (NH_4_)_2_SO_4_ 0.1 g, agar 20 g, distilled water 1,000 mL). After 15 days of incubation at 28°C, the appearance of a halo around the colony indicated phosphate solubilization. The phosphate solubilization ability was directly reflected by the halo diameter size; all experiments were carried out in triplicate.

### Disease Control of Red Pepper Anthracnose by GYUN-300 Bacterial Suspensions

An indoor experiment was conducted to examine the preventive and curative effects of GYUN-300 bacterial suspensions on anthracnose in wounded and non-wounded red pepper fruits (cv. Geochanghan). Fully ripened red pepper fruits, procured from the field were selected for the experiment. Fruits were surface sterilized in 70% ethanol for 3 min, immersed in 1% NaOCl for 1 min, and rinsed 2–3 times in SDW; the surface-sterilized red pepper fruits were air-dried at room temperature. Nearly five wounds were inflicted on the surface of fruits using a sterile needle, and one set of non-wounded red pepper fruits were also used in this experiment. To test for preventive effects, wounded or non-wounded fruits were treated with antagonistic bacterial suspensions of GYUN-300 (10^7^ CFU/mL) using a spray method (50 μL per fruit). Fruits treated with chemical pyraclostrobin and SDW served as positive and negative controls, respectively. After sufficient drying at room temperature, 10 μL of *C. acutatum* spore suspensions (10^5^ conidia/mL) were inoculated on wounded or non-wounded sites, dried at room temperature, then placed in plastic square plates (40 cm × 40 cm) containing filter paper under moist conditions. The disease index (DI), using the formula (DI = sum of disease ratings in fruits/total number of fruits assessed) was recorded 10 days after incubation at 25°C in comparison with a non-treated control, from which the control value (%) was calculated. To test for curative effects, wounded or non-wounded red pepper fruits were inoculated with 10 μL of *C. acutatum* spore suspensions (10^5^ conidia/ml), dried at room temperature, and placed in plastic square plates (40 cm × 40 cm) containing wet paper to maintain the humidity. Plates were incubated at 25°C for 10 days, treated with antagonistic bacterial suspensions, and incubated at 25°C for another 4 days; then the disease index, from which the control value (%) was recorded. Fruits treated with pyraclostrobin and SDW served as positive and negative controls, respectively.

### Effect of Antagonistic Bacteria on Growth in Red Pepper Seed Germination

Red pepper seeds (cv. Geochanghan) used for growth promotion were purchased from the local market. Seeds were washed with tap water to remove the coated chemical fungicides and then surface-disinfected with 1% NaOCl for 10 min; they were then rinsed in SDW and dried completely on filter paper. The bacterial inoculum was prepared by culturing on TSA plates for 3 days at 28°C, and cell suspensions were prepared at 10^6^ CFU/mL prior to inoculation. *Bacillus velezensis* AK-0 and *Serratia plymuthica* GYUN-8 were used as positive controls. They are commercially available in the market as biocontrol agents (BCAs), with names, Tangeokil and Serratan for AK-0 and GYUN-8, respectively in Korea. These BCAs are produced by Koreabio, Co. Ltd., South Korea; while seeds treated with SDW served as negative controls. Dried seeds were coated with bacterial cell suspensions (10^6^ CFU/mL) by immersing for 30 min, placed between double-layered wet papers, and incubated in a growth chamber at 25°C for 6 days. Then, seed germination (root length in mm) was measured and compared to that of the non-treated control; this experiment was repeated at least once.

### Growth Promotion Effect on Red Pepper Seedlings

To investigate the ability of GYUN-300 to promote growth, surface-sterilized red pepper seeds were sown in plastic trays (36 pots per tray) containing garden soil. After germination, 3-week-old red pepper seedlings were soil drenched with 25 mL of 3-days-old cultured bacterial suspensions (10^6^ CFU/mL) of GYUN-300, GYUN-8, or AK-0. Observations of growth promotion were recorded once every 7 days up to 40 days of the growing period; Seedlings were watered once every 3 days. The average length (cm) of seedlings was measured; this measurement was constrained to the length of the aboveground biomass. Four replications were used per treatment; each replication consisted of 20 seedlings under greenhouse conditions.

### Fungicidal Resistance Test to GYUN-300 Bacterial Suspensions

To investigate the fungicidal resistance to GYUN-300, 11 types of chemical fungicides currently used to control red pepper anthracnose in Korea were used (Supplementary Table 1). A single colony of GYUN-300 was inoculated into TSB and incubated under shaking conditions (180 rpm) at 28°C for 3 days. From this, 100 μL of the suspensions were spread onto freshly prepared TSA plates. Sterile paper discs (6 mm in diameter) impregnated with 10 μL of chemical fungicides at different concentrations (i.e., 0.5×, 1.0×, and 2.0×) were placed onto the TSA plates. Results were observed after 3 days of incubation at 28°C; the experiment was carried out in triplicate. Bacterial resistance to fungicides was determined based on the inhibition zone.

### Field Evaluation of the Application of GYUN-300 on Disease Suppression of Red Pepper Anthracnose

A field experiment was conducted to investigate the biological control of red pepper anthracnose using GYUN-300 antagonistic bacteria in Korea. At first, the seedlings were raised in plastic trays (36 pots per tray) by placing a single seed per pot. Three-week-old red pepper seedlings were transplanted to a field belongs to Andong National University, located at Andong, Gyeongbuk Province, South Korea. Seedlings were planted at a distance of 100 cm between rows and 40 cm between plants. After preparing the field, the soil was fertilized with NPK before starting the transplantation. The planting row was covered with non-woven fabric material for weed control. Antagonistic microorganism treatments were grouped into three: soil drenching, foliar spray, and foliar spray + soil drench. The GYUN-8 and AK-0 bacteria were used as positive controls, Pyraclostrobin was used as chemical control, and water treatment was served as non-treated control. Bacterial cell suspensions prepared at a concentration of 10^7^ CFU/mL were used for all treatments. The field was irrigated 10 days intervals. The first dose was administered on June 17, 2020, and a total of nine treatments were administered at 10 days intervals until August 31, 2020. The pyraclostrobin emulsion was used as chemical control, and tap water was used as negative control. The disease incidence (%) was calculated from the disease index ratings (Supplementary Table 2). Four replications were used per treatment; each replication consisted of 20 plants under field conditions.

### Statistical Analysis

The data were subjected to analysis of variance (ANOVA) using SAS JMP software ver. 3. (SAS Institute Inc, 1995). The experiments were set up as completely randomized block designs under field conditions. Significant differences between treatment means were determined using the least significant difference (LSD) at *p* < 0.05. All experiments were carried out at least twice; data were analyzed separately for each experiment. The results of one representative experiment are shown.

## Results

### *In vitro* Antagonistic Activity of GYUN-300

**FIGURE 1:**
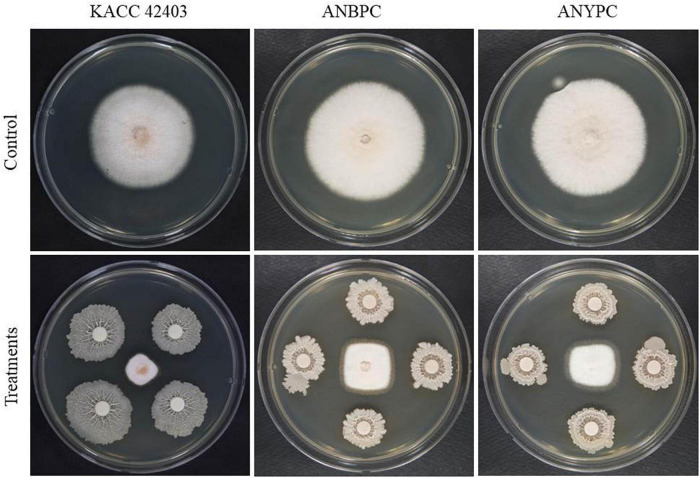
*In vitro* antagonistic activity of *Bacillus tequilensis* GYUN-300 on the inhibition of Colletotrichum *acutatum* fungal stains in comparison with the non-treated control. Mycelial growth of fungal pathogens on potatoes dextrose agar plates was recorded 7 days after incubation at 25°C. The experiment was repeated at least once, with each treatment carried out in replicate.

**FIGURE 2:**
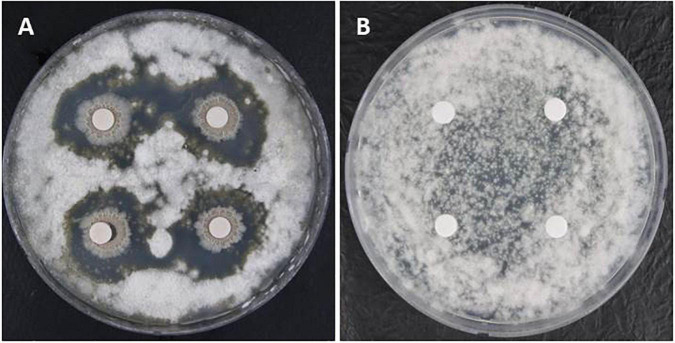
Dual culture plate assay for the *in vitro* inhibition of *Colletotrichum acutatum* mycelial growth using an antagonistic bacterium GYUN-300: **(A)** sterile paper discs loaded with bacterial suspensions (10^8^ CFU/mL) placed onto the potato dextrose agar (PDA) plates containing pathogenic fungal spores; and **(B)** sterile paper discs (6 mm in diameter) loaded with sterilized distilled water (SDW) served as the non-treated control. The inhibition zone was measured 7 days after incubation at 25°C. The experiment was repeated at least once, with each treatment carried out in triplicates (Petri dishes).

The GYUN-300 bacterium was tested for *in vitro* antagonistic activity against three fungal pathogenic isolates; *C. acutatum* KACC42403, ACPP014, and ACPP015, all of which cause anthracnose disease in red pepper fruits (Figure 1). The mycelial growth of all three pathogenic fungal isolates was inhibited at a greater level than the non-treated control. Among the three fungal isolates, the mycelial growth of isolate *C. acutatum* KACC42403 was greatly inhibited by GYUN-300 7 days after incubation at 25°C, when compared the growth inhibition of the other two pathogens. GYUN-300 was most effective with an average inhibition zone of 28, 25.3, and 25.5 mm against *C. acutatum* KACC42403, ACPP014, and ACPP015, respectively (Supplementary Table 3). The dual culture plate assay on the PDA medium exhibited greater inhibition of mycelial growth of *C. acutatum* KACC42403 on PDA plates 5 days after incubation at 25°C, while growth inhibition was not observed in the non-treated control (Figure 2).

### Effect of GYUN-300 Cell Suspensions or Culture Filtrate Treatment on Conidial Germination of *Colletotrichum acutatum* Under *in vitro* Conditions

**FIGURE 3:**
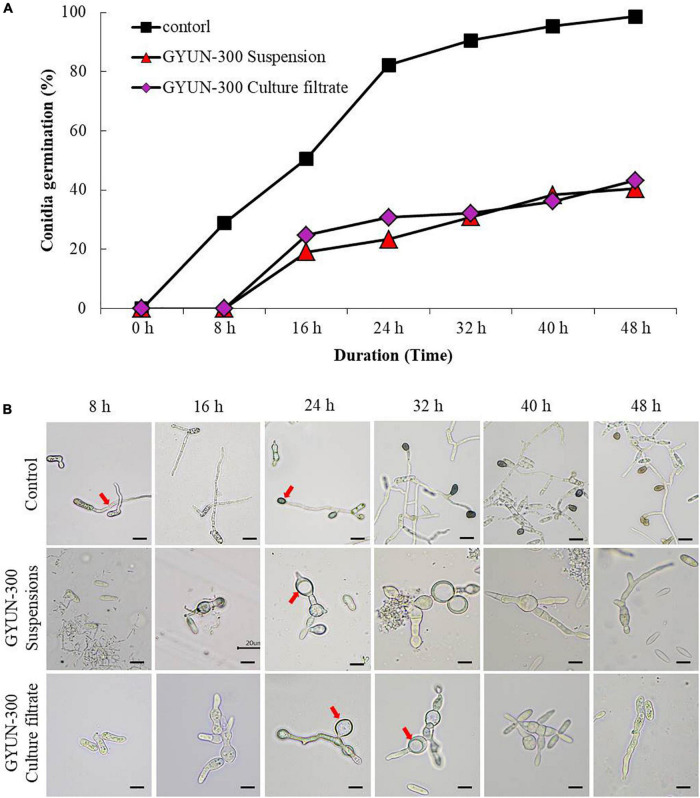
Effect of bacterial cell suspensions and culture filtrate (CF) of *Bacillus tequilensis* GYUN-300 treatment on conidia germination rate (%) of *Colletotrichum acutatum* and microscopic observation: **(A)** Conidial germination rate (%) was suppressed by CF and bacterial cell suspensions, while the germination rate (%) was increased in the non-treated control; and **(B)** microscopic observations of *C. acutatum* fungal spore germination after GYUN-300 treatment during the incubation period from 8 to 48 h. The germination counting was carried out using a hemocytometer. Bar = 10 μm. The experiment was repeated at least once, with treatments repeated in triplicate producing similar results.

When conidial spores of *C. acutatum* KACC42403 were treated with GYUN-300 bacterial cell suspensions (10^6^ CFU/mL) or their CF under *in vitro* conditions, there were different levels of damages in the conidia germination and germ tube lengths; these were compared to the non-treated control. Conidial spore germination analysis using a hemocytometer showed greater inhibition percentage of spores after 16 h incubation in GYUN-300 cell suspensions-treated conidia compared to CF-treated conidia. After 48 h of incubation, the conidial germination rate was 40 and 43% in the GYUN-300 cell suspension and its CF treatments, respectively (Figure 3A); while the spore germination increased drastically in the non-treated control. GYUN-300-treated conidia resting on the hard glass surface did not germinate, while from 16 h onward, water-treated conidia germinated and formed appressoria through germ tubes (Figure 3B). At 48 h, all conidia had germinated and appressoria were formed through germ tubes in the water-treated control; this did not occur with the GYUN-300-treated conidia. At 16 h itself, there was a suppression of conidial germination, resulting in gall-like formation on the hyphal wall without further leading to germination during 48 h of incubation. This result suggests that GYUN-300 cell suspensions played a role effectively in suppressing conidial germination of *C. acutatum* KACC42403 (Figure 3B).

### Inhibitory Effects of Volatile Organic Compounds Produced by GYUN-300 on *in vitro* Fungal Growth of *Colletotrichum acutatum*

**FIGURE 4:**
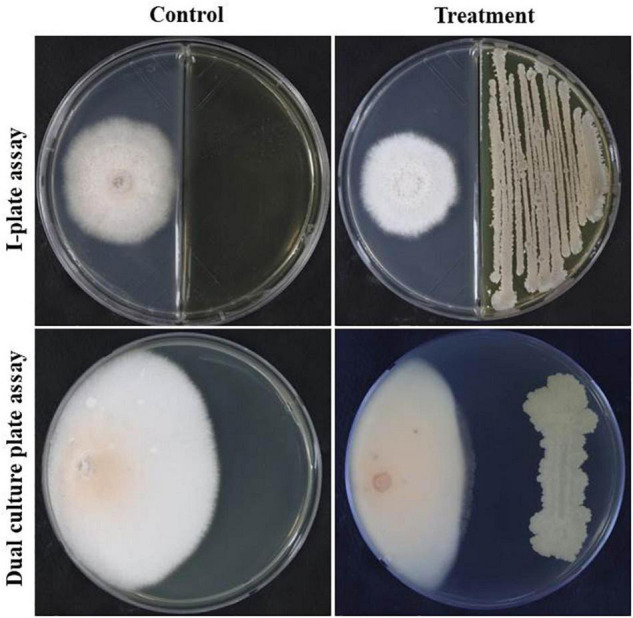
Inhibition of *Colletotrichum acutatum* mycelial growth by bacterial volatiles produced by GYUN-300 under *in vitro* conditions using the I-plate and dual culture plate assays. Growth inhibition was observed in the I-plate and dual culture plate assays by volatiles in comparison with the non-treated control.

We tested the effect of bacterial volatile organic compounds (VOCs) released by GYUN-300 cells on the inhibition of mycelial growth of *C. acutatum* KACC42403 under *in vitro* conditions using the I-plate or dual culture plate assays. Exposing *C. acutatum* KACC42403 to VOCs released by GYUN-300 bacterial suspensions significantly inhibited fungal mycelial growth 7 days after incubation at 25°C (Figure 4). When bacterial cells colonized the entire Petri dish, fungal growth had been drastically reduced by 20% greater in I-plate assay in comparison with the growth reduction of the same fungal pathogen in the dual culture plate assay. This was because the spread of the bacterial inoculum was relatively lower than that observed in the I-plate assay; the specific VOCs involved are yet to be determined.

### Morphological Analysis of *Colletotrichum acutatum* in the Presence or Absence of GYUN-300 Cell Suspensions

Distinct morphological characterization of the mycelial growth of *C. acutatum* in the presence or absence of GYUN-300 cell suspensions were carried out *in vitro* using a dual culture plate assay (Supplementary Figure 1). The mycelium grown in the presence of GYUN-300 was elongated, where hyphae were formed producing gall-like formations on the mycelia. This was considered deformed mycelia, and mycelial growth was healthy without any signs of deformation when it was grown without the presence of the GYUN-300 cell suspensions.

### Identification of Antagonistic Bacteria Using MicroLog System

The characteristics of GYUN-300 based on carbon source utilization and various chemical reagents assessed by the MicroLog system revealed that GYUN-300 was sensitive to various carbon sources, such as glycol-L-Proline, maltose, and D-fructose (Supplementary Table 4). By contrast, antibiotics, such as rifamycin SV, vancomycin, and minocycline, showed a negative reaction to the chemical substances (Supplementary Table 5). These traits were compared with *Bacillus subtilis*, where they were found to have a 57% probability of occurring compared with the existing database. The bacteria was identified as *Bacillus* sp., based on carbohydrate and chemical utilization, as this method identifies specific bacteria by matching with carbon utilization in the database.

### Molecular Identification of GYUN-300

The isolate GYUN-300 was characterized using 16S rDNA gene sequencing. Comparison of the specific sequence of the ribosomal gene with sequences deposited in GenBank (accession no. OK001770) suggest that the GYUN-300 isolate belonged to the *Bacillus* genus, and shared the highest homology with *B. tequilensis* (99.93%). In the phylogenetic tree, GYUN-300 was clustered with other *Bacillus* spp. and was closely related to *B. tequilensis* (Supplementary Figure 2); thus, molecular characteristics confirmed this species as *B. tequilensis*. In the phylogenetic tree, the isolate was clustered with other *Bacillus* species that was closely related to *B. tequilensis*.

### GYUN-300 Whole-Genome Sequence Analysis and Annotation

**FIGURE 5:**
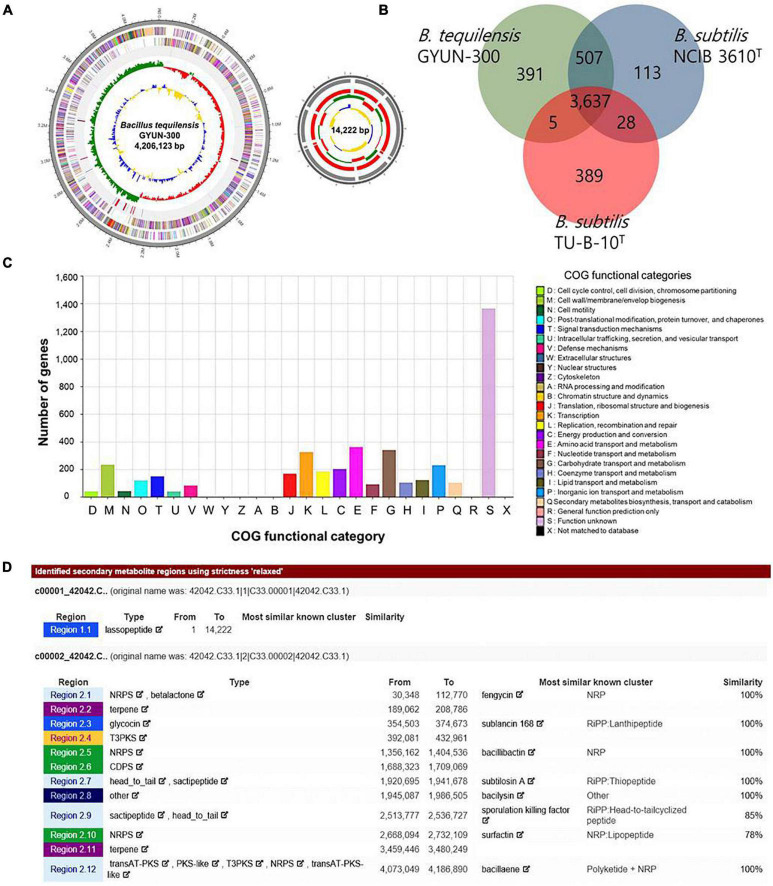
Whole-genome map of *Bacillus tequilensis* GYUN-300 and annotation. **(A)** Marked characteristics shown from the outside to the center; coding sequence (CDS) on forward strand, CDS on reverse strand, tRNA, rRNA, guanine-cytosine (GC)-content, and GC skew. CDS genome consists of a single circular chromosome that is 4.2 mb in size, and represents one contig from the outer part of the circle; the second circle represents forward, the third represents reverse strain, and the fourth circle represents tRNA and rRNA positions; **(B)** distribution of orthologous genes in the *Bacillus tequilensis* GYUN-300, *B. subtilis* subsp. *subtilis* NCIB 3610*^T^*, and *B. subtilis* subsp. *spizizenii* TU-B-10*^T^* genomes. The Venn diagram shows the summary of unique SNPs from total genes of the *B. tequilensis* GYUN-300 strain. This analysis exploits all CDS of the genomes and is not restricted to the core genome; **(C)** the clusters of orthologous genes (COG) function annotation of *B. tequilensis* GYUN-300, and distribution of genes in different COG function categories; and **(D)** analysis of secondary metabolites of GYUN-300 using the antiSAMASH program.

PacBio RSII NGS equipment was used at a sequencing depth of 179.34× to obtain complete sequence data for GYUN-300. The generated raw data were assembled using the HGAP2 protocol to obtain a FASTA file consisting of one contig. In genome analysis, GYUN-300 was identified as *B. tequilensis*, where its core genome coverage was 96.7%. The genome of the *B. tequilensis* GYUN-300 (GYUN-300) strain consisted of a circular chromosome of 4,220,345 bp, with 4,789 predicted protein-coding sequences (CDSs), 38 rRNA genes, 86 rRNA genes, and an average G + C content of 43.6% based on the NCBI Prokaryotic Genomes Automatic Annotation Pipeline (PGAAP) analysis (Figure 5A and Table 1); the full genome sequence was deposited in NCBI under the GenBank accession number OK001770. Comparative analysis between the genome sequences of GYUN-300 and four other *Bacillus* strains showed a similar genome (Table 1). The genome of the GYUN-300 strain was compared with the four closest known evolutionary relatives: *B. subtilis* subsp. *subtilis* NCIB 3610, *B. subtilis* subsp. *spizizenii* TU-B-10, and *B. subtilis* subsp. *inaquosorum* KCTC 13429. Most genes in GYUN-300 were associated with secondary metabolite biosynthesis, transport, amino acid metabolism, carbohydrate metabolism, and catabolism. The first outer circle in the gray of the genome map represents one contig, the second circle represents the forward, the third circle represents the CDSs on the reverse strand, and the fourth circle indicates the tRNA and rRNA positions (Figure 5A). In addition to the gDNA, it was determined that the genome was likely to have an additional plasmid form. The fifth circle represents the GC skew, used as a reference point; values higher than this are indicated in green, while lower values are indicated in red. The sixth circle is GC ratio metric, where values greater than the average GC ratio, were expressed in blue, and lower values were represented in yellow; the GC skew and GC ratio were expressed at 10 kb intervals.

**TABLE 1:** Genomic features of the *Bacillus tequilensis* GYUN-300 genome and comparison with genomes of other *Bacillus* subsp. belonging to the *Bacillus subtilis* group.

Taxon name	Strain name	No. of contigs	Genome size	DNA G + C content (%)	No. of CDSs	No. of rRNA genes	No. of tRNA genes
*B. tequilensis*	GYUN-300	2	4 220 345	43.6	4,789	38	86
*B. subtilis* subsp. *subtilis*	NCIB 3610*^T^*	2	4 299 822	43.3	4,329	30	88
*B. subtilis* subsp. *spizizenii*	TU-B-10*^T^*	1	4 207 222	43.8	4,144	30	92
*B. subtilis* subsp. *inaquosorum*	KCTC 13429*^T^*	1	4 350 498	43.8	4,280	27	86

Venn diagrams (Figure 5B) compare the common CDSs among *B. tequilensis* GYUN-300, *B. subtilis* subsp. *subtilis* NCIB 3610, and *B. subtilis* subsp. *spizizenii* TU-B-10. This section summarizes the unique protein-encoding genes from the total genes of GYUN-300 is presented here. There were 3637 common high-expression gene families shared among GYUN-300, NCIB 3610, and TU-B-10 (Figure 5B), while 391, 113, and 389 genes were determined to be unique for GYUN-300, NCIB 3610, and TU-B-10, respectively. All predicted CDSs of GYUN-300 were compared in the clusters of orthologous genes (COG) database to identify homologous amino acid sequences. Each functionally annotated protein was assigned a COG number, representing a class of proteins; then, proteins were subjected to functional clustering analysis according to the COG function (Figure 5C). The detection of secondary metabolites from GYUN-300 was analyzed using antiSMASH (Figure 5D). A total of 12 biosynthetic gene clusters were detected in GYUN-300. Clusters 1, 3, 5, 7, and 12 showed 100% similarity to fengycin, sublancin 168 A, bacillibactin, subtilosinA, and bacillaene, respectively; cluster 10 showed 78% similarity to surfactin.

### Production of Lytic Enzymes, Siderophore Production, and Phosphate Solubilization by Antagonistic Bacteria

The antagonistic bacterium, GYUN-300, was considered for the production of hydrolyzing enzymes, such as cellulase, protease, and amylase, under *in vitro* conditions (Supplementary Figure 3A). GYUN-300 was found to produce protease at a greater level with an average inhibition zone of 25 mm. By contrast, other enzymatic activities, such as cellulase and amylase, had inhibition zones of 18 and 23 mm, respectively. GYUN-300 displayed strong inhibitory activity against fungal pathogens and showed strong amylase activity (Supplementary Figure 3A). There was greater production of all three enzymes (i.e., cellulase, protease, and amylase) by GYUN-300 compared with the non-treated control (SDW); this was in terms of inhibition zones around inoculation sites on the solid media in Petri dishes through the degradation of various substrates, such as CMC, proteins, and soluble starch, respectively.

The capacity of GYUN-300 to produce siderophores was qualitatively observed using a CAS assay. The positive reaction of the qualitative assay results in a color change in the CAS agar medium from blue to orange around the inoculation site (Supplementary Figure 3B). The color changed from blue to light orange 4 days after incubating the plates at 28^°^C, while there was no color development around the non-treated control (SDW). This result suggests that siderophores produced by this bacterium helped to obtain dissolved iron by binding to Fe^3+^; this enables competition with pathogens for available iron, which is essential for survival. The phosphate solubilization ability of the bacterium GYUN-300 was determined on tricalcium phosphate medium, where GYUN-300 solubilized the inorganic phosphate at a moderate level. This converted phosphorous into an available form, which is important as it is one of the major essential macronutrients for plant growth and development (Supplementary Figure 3B); there was no growth inhibition in the water-treated control.

### Control of Red Pepper Anthracnose by GYUN-300 Bacterial Suspensions

**FIGURE 6:**
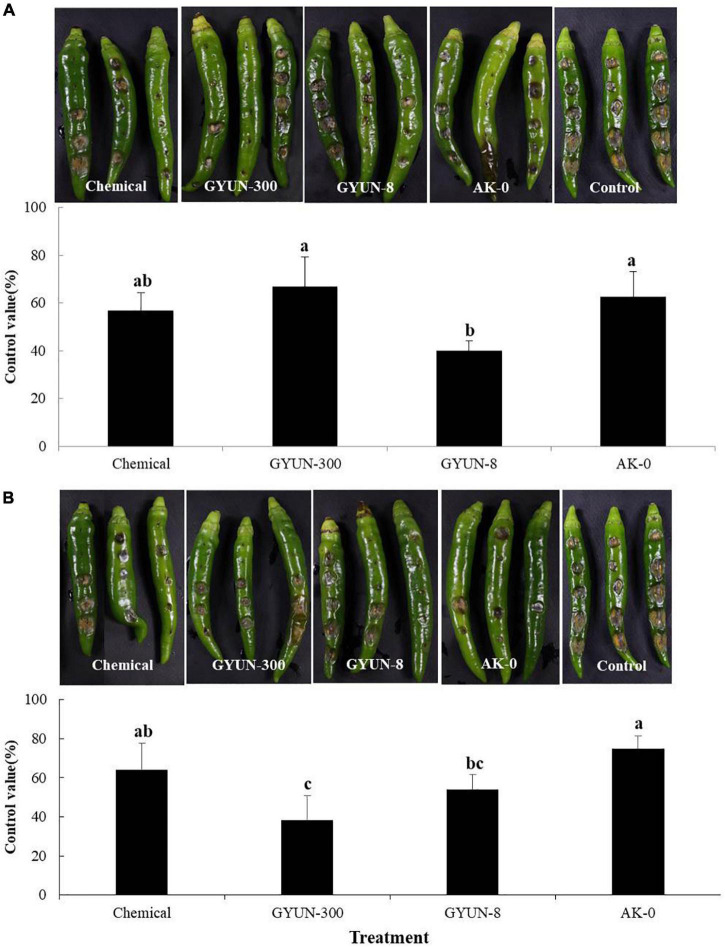
Protective and curative effects of GYUN-300 on disease suppression of red pepper anthracnose in wounded fruits. **(A)** For protective effects, bacterial suspensions (10^7^ CFU/mL) were sprayed on the detached green pepper fruits, followed by inoculation with *Colletotrichum acutatum* conidia suspensions (10^5^ conidia/mL) on the same day. The disease severity (%) was recorded 10 days after incubation at 25^°^C. **(B)** For curative effects, symptom development was observed 10 days after pathogen inoculation with *C. acutatum* conidia suspensions (10^5^ conidia/mL) on detached green pepper fruits, followed by treatment with bacterial suspensions (10^7^ CFU/mL) using a spray method. Disease severity (%) was recorded 10 days after incubation at 25^°^C. The experiment was carried out two times with six replicates (fruits) per treatment, showing similar results. Bars with the same letters do not differ significantly between each other according to the least significant difference (LSD; *p* < 0.05).

**FIGURE 7:**
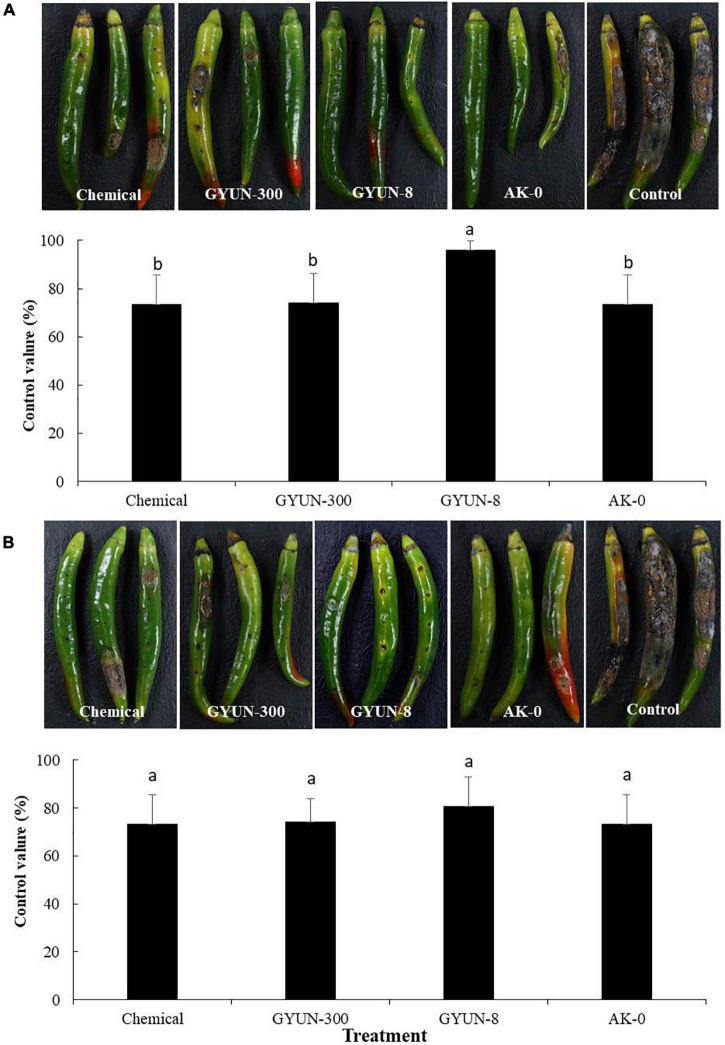
Protective and curative effects of GYUN-300 on disease suppression of red pepper anthracnose in non-wounded fruits. **(A)** For protective effects, bacterial suspensions (10^7^ CFU/mL) were sprayed on the detached green pepper fruits without wounding, followed by inoculation with *Colletotrichum acutatum* conidia suspensions (10^5^ conidia/ml) on the same day. Disease severity (%) was recorded 14 days after incubation at 25^°^C. **(B)** For curative effects, symptoms were observed 14 days after pathogen inoculation with *C. acutatum* conidia suspensions (10^5^ conidia/mL) on detached green pepper fruits without wounding, followed by treatment with bacterial suspensions (10^7^ CFU/mL) using a spray method. Disease severity (%) was recorded 10 days after incubation at 25^°^C. The experiment was carried out two times with six replicates (fruits) per treatment, showing similar results. Bars with the same letters do not differ significantly between each other according to the least significant difference (LSD; *p* < 0.05).

For anthracnose disease control by GYUN-300 bacterial suspensions, the red pepper fruits were treated with conidia suspensions on wounded or non-wounded fruits. In terms of preventive effects, where fruits were inoculated with pathogens after treatment with bacterial suspensions, the GYUN-300 bacterial treatment significantly (*P* < 0.05) controlled the anthracnose by 66.6%. This was higher than the chemical, GYUN-8, and AK-0 treatments, where they controlled the anthracnose by 56.6, 40, and 62.5%, respectively (Figure 6A). By contrast, in terms of the curative effect of wounded red pepper fruits, the GYUN-300 treatment controlled the anthracnose by 38.3%; this level of control was lower than the other treatments (Figure 6B). GYUN-300 treatment by the preventive method was better than the curative effect in wounded red pepper fruits. When GYUN-300 treatment was tested on non-wounded red pepper fruits, there was no significant difference in the control value among the three treatments. However, GYUN-8 treatment exhibited a greater control value (95.8%) than the other three treatments in terms of its protective effect (Figure 7A). In terms of the curative effects, the disease control using the chemical, GYUN-300, and AK-0 treatments was the same as that for the protective effects of non-wounded red pepper fruits; however, the GYUN-8 treatment exhibited less control than its protective effect (Figure 7B). The level of disease control with the non-treated control was zero in all treatments. These results suggest that the GYUN-300 strain is more effective as a preventive method compared to its curative effect.

### Effect of GYUN-300 Treatment on Seed Germination and Growth Promotion in Red Pepper

A double-layered wet paper method was used to evaluate the growth promoting effect of GYUN-300 on red pepper seed germination under *in vitro* conditions. The average length of seedlings was higher in the AK-0 treated seedlings than other treatments (Supplementary Figure 4A). However, seedling length in GYUN-300 treated seedlings was higher than the non-treated control and GYUN-8-treated seedlings. The average seedling lengths were 31.7, 29.2, 39.6, and 25.3 mm, in the GYUN-300, GYUN-8, AK-0 treatments, and control, respectively. Similarly, the plant growth-promoting ability of GYUN-300 on red pepper seedlings was greater in terms of plant height compared to that in the non-treated control and other treatments, such as GYUN-8 and AK-0 (Supplementary Figure 4B); this indicates that GYUN-300 may be used for growth promotion.

### Fungicidal Resistance to the Bacterium GYUN-300

The GYUN-300 strain was tested for resistance against various chemical fungicides under *in vitro* conditions. The bacterium GYUN-300 showed resistance against nine of the ten chemical fungicides tested in this study; while the bacterium showed sensitive to the fungicide Acibenzolar-S-methyl/Chlorothalonil, which inhibited bacterial growth on the TSA plates (Supplementary Table 6). This result suggests that most chemical fungicides used to control red pepper anthracnose do not affect GYUN-300 cell growth. These results suggest that GYUN-300 cells may be used synergistically with the chemical fungicides to control plant disease.

### Effect of GYUN-300 on Disease Control of Red Pepper Anthracnose Under Field Conditions

**FIGURE 8:**
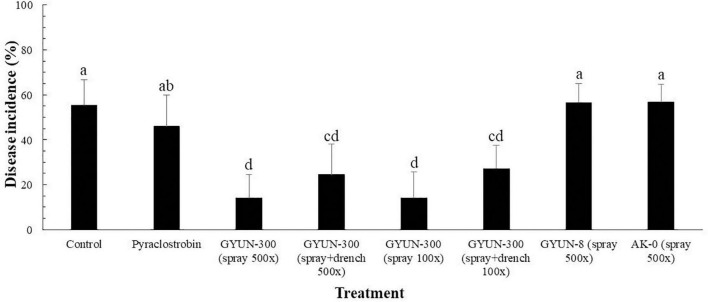
Effect of GYUN-300 treatment on disease suppression of anthracnose caused by *Colletotrichum acutatum* under field conditions. Various treatments of GYUN-300 were compared with the chemical controls and non-treated control. Bars with the same letters do not differ significantly between each other according to the least significant difference (LSD; *p* < 0.05).

Based on the *in vitro* results of disease suppression by GYUN-300, we tested the ability of GYUN-300 bacterial cells to suppress red pepper anthracnose caused by *C. acutatum* under field conditions. There was greater reduction in disease incidence (%) in the GYUN-300 treatment by foliar spray in comparison with the soil drench or combined application of soil drench + foliar spray (Figure 8). The disease incidence (%) was recorded as only 14% at both concentrations (500× and 100×) of GYUN-300 cell suspensions using a foliar spray method, while the disease incidence was 24.5% by combined method of spray + soil drench. On the other hand, the disease incidence was found at a greater level (56%) in both positive controls GYUN-8 and AK-0 by foliar spray method. Whereas, the disease incidence in chemical control (Pyraclostrobin) was observed as 46.1%, this is better than positive controls and non-treated control. Overall, all treatments with GYUN-300 showed better performance in controlling disease incidence (%) compared with the chemical treatments and non-treated control. These results suggest that GYUN-300 is a potential candidate for the biological control of red pepper anthracnose in Korea.

## Discussion

*Bacillus* species are widespread in and produce numerous antibiotic compounds that are active against several phytopathogens as an alternative to chemical fertilizers and synthetic pesticides (Shahid et al., 2021). In this study, we isolated the *B. tequilensis* GYUN-300 strain from fresh sanghwang mushroom, as it exhibits potent antagonistic activity against red pepper anthracnose caused by *C. acutatum* under *in vitro* and field conditions. Previously, several *Bacillus* species have demonstrated antagonistic activity against several *Colletotrichum* species, including *C. acutatum* (Han et al., 2015; Caulier et al., 2019).

This study describes the isolation, identification, and characterization of an antagonistic *Bacillus* sp. isolated from an edible mushroom. GYUN-300 was confirmed as *B. tequilensis* based on its morphology and the BIOLOG program results. Furthermore, the GYUN-300 strain was characterized by molecular analysis using 16S rDNA sequencing; this strain was identified as *B. tequilensis* GYUN-300. The GYUN-300 strain showed 99.9% homology with the reported database strains of other *B. tequilensis*. Anthracnose from *Colletotrichum* may cause considerable damage to various crops, including red pepper (Than et al., 2008; Kim et al., 2010); *Colletotrichum* spp. are frequently associated with several pepper diseases worldwide (Liu et al., 2016). There are limited reports on microbial-based control of anthracnose disease, and to the best of our knowledge, this is the first report on the use of the GYUN-300 as a potential BCA for red pepper anthracnose; this is also the first analysis of the whole-genome sequencing of GYUN-300. The results provide an understanding of the biocontrol mechanism of the GYUN-300 strain, providing a possible alternative BCA to control red pepper anthracnose in Korea.

It was found that treatment with GYUN-300 CF was equally effective as the cell suspensions in inhibiting the conidial germination of *C. acutatum* under *in vitro* conditions. Similarly, a recent study by Choub et al. (2021b) reported that the CF from *B. velezensis* CE100 exhibited antifungal activity against *Colletotrichum gloeosporioides* through the production of cyclic tetrapeptides from the CF. Another study reported that the CF from *B. subtilis* inhibited the growth and spore germination of *C. gloeosporioides* through mycolytic enzyme-mediated antagonism (Ashwini and Srividya, 2014). Recently, Sarwar et al. (2018) reported that the growth of various phytopathogens, such as *Fusarium moniliforme*, *Fusarium oxysporum*, *Fusarium solani*, and *Trichoderma atroviride*, was suppressed by *Bacillus* spp. through the production of a broad spectrum of lipopeptide biosurfactants. This is supported by recent reports (Jayapala et al., 2019; Wu et al., 2019), which have demonstrated that *Bacillus* spp. played a role in producing antibiotic compounds to protect red pepper from anthracnose and wilt diseases caused by *Colletotrichum capsici* and *Rhizoctonia solani*, respectively. Zalila-Kolsi et al. (2016) reported that a few *Bacillus* species isolated from wheat rhizosphere soil exhibited *in vitro* antagonistic activity against several phytopathogenic fungi, including *F. graminearum*, which causes durum wheat disease.

The microscopic observations in this study indicate that the suppressive effects of bacterial suspensions of GYUN-300 and its CF may be due to antibiosis mechanisms. The GYUN-300 strain affected the conidial germ tube and mycelial morphology of *C. acutatum* KACC42403, causing severe damage to the fungal hyphae. Numerous studies have demonstrated the antifungal mechanisms of soluble non-volatile bioactive compounds, such as lipopeptides and proteins, produced by various *Bacillus* species against several soil-borne diseases (Zhang et al., 2020). Conversely, the suppression of mycelial growth of fungal phytopathogens caused by bacterial antagonists has been attributed to VOCs, involved in biocontrol interactions by *Bacillus species* against fungal pathogens, such as *Botrytis cinerea* (Sharifi and Ryu, 2016), *Penicillium expansum*, *Alternaria alternata* (Gao et al., 2018), and *C. gloeosporioides* (Chowdhury et al., 2015).

The whole-genome sequence of GYUN-300 was characterized; this consisted of a 4.22 Mb chromosome, and this genome was compared to other *Bacillus* species (Table 1). In particular, biocontrol-related genes and gene clusters involved in the antibiotic may produce differences in biocontrol targets and efficacy between GYUN-300 and other *Bacillus subtilis* strains. These results suggest that all other strains may prevent the host plant from the disease by genes involved in the synthesis of secondary metabolites and exhibit strong antifungal activities against various fungal phytopathogens (Pusztahelyi et al., 2015; Costa et al., 2019). *Bacillus* species produce cyclic lipopeptides (CLPs), known for their broad-spectrum antagonistic activity mediated by secondary metabolites (Kim et al., 2021). This may have contributed to antagonistic activity against *C. acutatum*. Whole-genome comparisons revealed that the GYUN-300 strain showed high similarity to *B. subtilis* subsp. *subtilis* NCIB 3610*^T^*, *B. subtilis* TU-B-10*^T^*, and *B. subtilis* subsp. *inaquosorm* KCTC 13429*^T^* strains currently used as BCAs in field conditions (Zhang et al., 2016; Gu et al., 2021). The COG database was used to functionally categorize predicted proteins from the distribution of genes (Galperin et al., 2015), and the COG categories were compared among the four strains, including GYUN-300. The COGs of the four strains showed highly similar distributions, suggesting that these strains have comparable biological characteristics. A total of 12 putative biosynthetic gene clusters for secondary metabolites were detected in the GYUN-300 genome. Some are involved in the metabolism of amino acids, carbohydrates, lipid transport and metabolism, and catabolism of secondary metabolite biosynthesis (Zhang et al., 2016). These functions are important for antagonistic agents against various phytopathogens, including *C. acutatum*, which causes red pepper anthracnose. The genome of GYUN-300 was investigated for the presence of gene clusters involved in the biosynthesis of CLPs that were predicted by antiSMASH analysis; this is a web server and stand-alone tool for the automatic genomic identification and analysis of biosynthetic gene clusters.^3^

Furthermore, the GYUN-300 strain produced hydrolytic enzymes, such as cellulase, protease, amylase, and siderophores, as well as phosphate solubilization, which are common features of antagonistic bacterial isolates (Belbahri et al., 2017). Siderophores are powerful ferric iron-chelating molecules produced by microorganisms that acquire iron, which is essential for crop growth. Siderophores differ from one another in their chemical structure and properties (Ahmed and Holmström, 2014), and have been suggested as environment-friendly alternatives to hazardous pesticides (Schenk et al., 2012). Siderophore-producing bacteria improve iron nutrition and slow down pathogen growth by limiting iron availability for pathogens (Nabila and Kasiamdari, 2021). However, the development of fungicide resistance in antagonistic bacteria is an increasing threat in plant disease management (Alizadeh et al., 2020). At times, the antagonistic bacteria in combination with agrochemicals are effective in suppressing pathogen growth at a greater level when compared with individual treatments under field conditions (Ons et al., 2020). This is in agreement with the results from this study, suggesting that GYUN-300 cell suspensions may be used in synergy with chemical fungicides.

The red pepper anthracnose disease was suppressed by GYUN-300 at a greater level in comparison with other positive controls, such as GYUN-8 and AK-0, and chemical control under field conditions. Similarly, a recent study by Choub et al. (2021a) reported that the field application of *B. velezensis* CE 100 culture broth resulted in a 1.3-fold and 6.9-fold decrease in anthracnose disease severity on walnut trees compared to the conventional and control groups, respectively. Previously, Park et al. (2001) reported to suppress the anthracnose disease in cucumber by the application of *Bacillus amyloliquefaciens* EXTN-1, and another strain *Bacillus vallismortis* BS07 suppressed the disease incidence (%) of anthracnose caused by *C. acutatum* on red pepper fruits at a greater level in comparison with the chemical control under field conditions (Park et al., 2013), which is in agreement with our results. Additionally, our results are supported by a recent study (Prihatiningsih et al., 2019), which displayed the control of red pepper anthracnose using microencapsulated *B. subtilis* B298 by spray method under field conditions. On the other hand, recently our team (Kim et al., 2021) reported to show the disease control of anthracnose caused by *C. gloeosporioides* on fresh apples using a biocontrol agent *B. velezensis* AK-0.

In conclusion, the GYUN-300 strain isolated from the edible sanghwang mushroom exhibited significant antagonistic activity against red pepper anthracnose caused by *C. acutatum*. The strain was identified as *B. tequilensis* GYUN-300 based on whole-genome sequence analysis. The strain exhibits *in vitro* antagonistic activity and suppression of conidial germination and *in planta* disease control of anthracnose. GYUN-300 suspension treatment has also demonstrated to control red pepper anthracnose in field conditions in Andong, Gyeongbuk Province, South Korea. Whole-genome sequencing revealed that the core genome of GYUN-300 was very similar to various *B. subtilis* strains. The differences between the various *B. tequilensis* strains in terms of control targets and efficacies may be attributed to variations in genes or gene clusters responsible for the biocontrol mechanism. Several CLP products of this strain were determined by analyzing secondary metabolite BGCs using antiSMASH software. GYUN-300 possessed several biosynthetic compounds, which may play an important role in controlling red pepper anthracnose through a mechanism of antibiosis. Overall, the results of this study indicate that GYUN-300 shows as an eco-friendly BCA against phytopathogens. Future research should investigate the signaling pathways involved in the antagonistic effects of secondary metabolites against pathogenic fungi.

## Data Availability Statement

The datasets presented in this study can be found in online repositories. The names of the repository/repositories and accession number(s) can be found in the article/Supplementary Material.

## Author Contributions

H-TK and YL designed the experimental setup and performed laboratory experiments. JK involved in the field experiment. KB and HK analyzed the data and wrote the manuscript. YJ supervised the project. All authors have reviewed and approved the manuscript.

## Conflict of Interest

The authors declare that the research was conducted in the absence of any commercial or financial relationships that could be construed as a potential conflict of interest.

## Publisher’s Note

All claims expressed in this article are solely those of the authors and do not necessarily represent those of their affiliated organizations, or those of the publisher, the editors and the reviewers. Any product that may be evaluated in this article, or claim that may be made by its manufacturer, is not guaranteed or endorsed by the publisher.

## Footnotes

1
https://kostat.go.kr

2
http://eggnogdb.embl.de

3
http://antismash.secondarymetabolites.org
