# Supplementary material for: Identification and Characterization of Bacillus tequilensis GYUN-300: An Antagonistic Bacterium Against Red Pepper Anthracnose Caused by Colletotrichum acutatum in Korea
Source: Front Microbiol. 2022 Mar 2;13:826827. doi: 10.3389/fmicb.2022.826827 (PMC8924438; doi:10.3389/fmicb.2022.826827)
Supplement: Supplementary file 1 [file Data_Sheet_1.docx]

**Supporting Information**

**Identification and characterization of *Bacillus tequilensis* GYUN-300: an antagonistic bacterium against red pepper anthracnose caused by *Colletotrichum acutatum* in Korea**

Hyeok-Tae Kwon^1^, Younmi Lee^1,2^, Jungyeon Kim^1^, Kotnala Balaraju^1,2^, Heung Tae Kim^2^, Yongho Jeon^1^*

^1^Department of Plant Medicals, Andong National University, Andong 36729, Republic of Korea

^2^Agricultural Science & Technology Research Institute, Andong National University, Andong 36729, Republic of Korea

^3^Department of Plant Medicine, Chungbuk National University, Cheongju 361-763, Republic of Korea

***Correspondence:**

Prof. Yongho Jeon

E-mail: [yongbac@andong.ac.kr](mailto:yongbac@andong.ac.kr); Tel: Tel: +82-54-820-5507; Fax: +82-54-820-6320

Supplementary Tables

Supplementary Table 1. List of fungicides tested for resistance against the antagonistic bacterium, GYUN-300

| Active ingredient | Concentration (%) | Formulation^a^ |
| --- | --- | --- |
| Azoxystrobin | 21.7 | SC |
| Acibenzolar–S-methyl/Chlorthalonil | 1.41 / 44.5 | SC |
| Acibenzolar–S-methyl/Dithianon | 1.75 / 30 | SC |
| Benomyl | 50 | WP |
| Kresoxim-methyl | 50 | WD |
| Pyraclostrobin | 22.9 | EC |
| Trifloxystrobin | 50 | WD |
| Tebuconazol | 25 | EC |
| Tebuconazol | 20 | SC |

^a^; SC; Suspension concentrate, WP; water powder, WD; water dispersible, EC; emulsified concentrate.

**Supplementary Table 2**. Anthracnose disease index on a red pepper plants

| **Disease index** | **Degree of diseased lesion** |
| --- | --- |
| 0 | No lesions on a plant |
| 1 | Diseased fruits ranging from 1 to 10% |
| 2 | Diseased fruits ranging from 10 to 25% |
| 3 | Diseased fruits ranging from 25 to 50% |
| 4 | Diseased fruits above 50% |

**Disease incidence (%)** = ∑ (Number of Disease plants X Disease score) / Total number of plants observed × Maximum score) × 100

**Supplementary Table 3.** Inhibition zone of *C. acutatum* mycelial growth from different locations by selected antagonistic bacterium isolated from sanghwang mushroom.

| Antagonistic microorganism | Pathogenic fungal mycelia (mm)^a^ | | |
| --- | --- | --- | --- |
|  | KACC42403 | ACPP014 | ACPP015 |
| Control | 53.3±0.5 | 56.3±0.4 | 57.4±0.3 |
| GYUN-300 | 28±2.1 | 25.3±0.4 | 25.5±1.6 |

^a^Inhibition zone was measured 7 d after incubation at 25 °C on PDK medium

Supplementary Table 4. Characteristic of GYUN-300 based on carbon source utilization assessed by the MicroLog system

| **Carbon source** | **GYUN-300** | ***B. subtilis*** |
| --- | --- | --- |
| Dextrin | + | + |
| α-D-lactose | - | - |
| D-mannose | + | + |
| D-mannitol | + | + |
| Glycol-l-proline | + | + |
| D-Galacturonic acid | + | + |
| Pyruvic acid methyl ester | + | + |
| g-Amino-n-butyric acid | - | + |
| Maltose | + | + |
| D-melibiose | + | + |
| D-fructose | + | + |
| L-arabitol | - | - |
| L-alanine | + | + |
| L-galactonic acid-g-lactone | + | - |
| D-lactic acid methyl ester | + | + |
| α-Hydroxybutyric acid | - | - |
| Gentiobiose | + | + |
| N-acetyl-d-glucosamine | + | + |
| D-glucose-6-phosphate | - | - |
| L-glutamic acid | + | + |
| Glucuronamide | - | + |
| α-Ketoglutaric acid | - | - |
| Acetoacetic acid | + | + |
| Sucrose | + | + |
| N-acetyl-d-mannosamine | + | + |
| L-fucose | - | + |
| D-fructose-6-phosphate | - | + |
| L-histidine | + | + |
| Mucic acid | + | + |
| D-malic acid | - | - |
| propionic acid | - | - |
| D-turanose | + | + |
| N-acetyl-d-galactosamine | - | + |
| L-rhamnose | + | + |
| D-aspartic acid | + | + |
| L-pyroglutamic acid | - | + |
| Quinic acid | + | - |
| Stachyose | + | - |
| N-acety-lneuraminic acid | - | - |
| Inosine | - | - |
| D-serine | - | - |

**-: Negative, +: Positive**

**Supplementary Table 5.** Metabolic activity of GYUN-300 at 24 h in the BIOLOG GENIII microplate assay

| **Substrates** | **GYUN-300** | ***B. subtilis*** |
| --- | --- | --- |
| Ph6 | + | + |
| Ph5 | + | + |
| 1% NaCl | + | + |
| 4% NaCl | + | + |
| 8% NaCl | + | + |
| 1% sodium lactate | + | + |
| Fusidic Acid | - | - |
| D-Serine | - | - |
| Troleandomycin | - | - |
| Rifamycin SV | - | + |
| Minocycline | - | - |
| Niaproof4 | - | - |
| Vancomycin | - | - |
| Tetrazolium violet | + | - |
| Tetrazolium blue | - | + |
| Nalidixic Acid | - | - |
| Lithium chloride | + | + |
| Potassium tellurite | + | + |
| Aztreonam | + | + |
| Sodium butyrate | + | + |
| Sodium bromate | + | + |
| Positive Control | + | + |

Supplementary Table 6. *In vitro* fungicidal sensitivity test against antagonistic GYUN-300 bacterium

| Fungicide | 0.5 $\boldsymbol{\times}$ | 1 $\boldsymbol{\times}$ | 2 $\boldsymbol{\times}$ |
| --- | --- | --- | --- |
| Azoxystrobin | - | - | - |
| Acibenzolar–S-methyl/Chlorthalonil | +++ | +++ | +++ |
| Acibenzolar–S-methyl/Dithianon | - | - | - |
| Benomyl | - | - | - |
| Kresoxim-methyl | - | - | - |
| Pyraclostrobin | - | - | - |
| Trifloxystrobin | - | - | - |
| Tebuconazol | - | - | - |
| Tebuconazol | - | - | - |

`*-: 0, +: 1~5mm, ++: 6~11mm, +++: 12mm.

**Supplementary Figures**


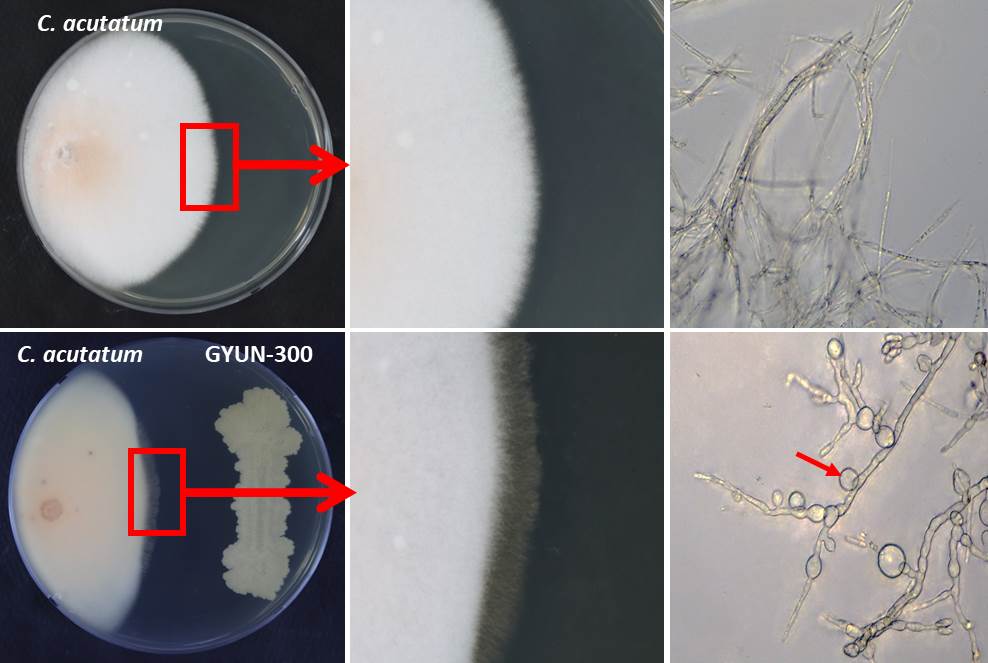


**Figure S1.** Distinct morphological characterization of mycelial growth of *C. acutatum* in the presence or absence of bacterial suspensions of GYUN-300 in dual culture plate assay.


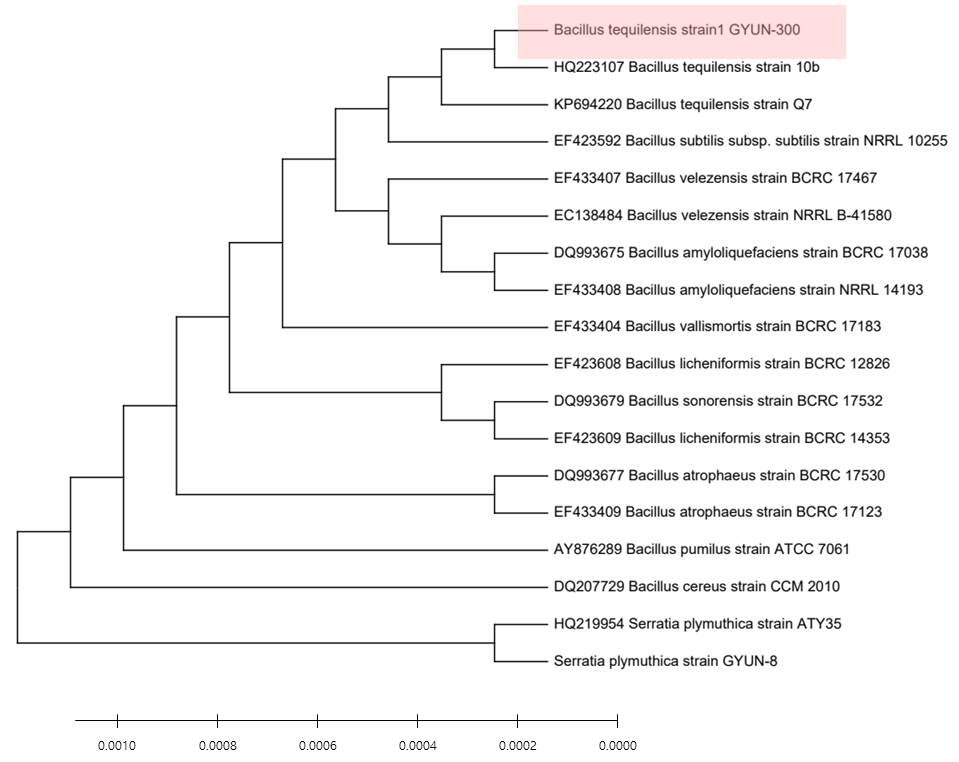


**Figure S2. Phylogenetic dendrogram constructed from comparative analysis of 16S rRNA gene sequences showing the relationships between *B. tequilensis* strain GYUN-300 and related *Bacillus* species.** Bootstrap values (expressed as percentages of 1000 replications) > 50% are shown at branch points and the species names are followed by the GenBank accession numbers. Maximum parsimony phylogenetic tree generated by the MEGA 4.0 program. The scale bar indicates 0.02 substitutions per nucleotide position.


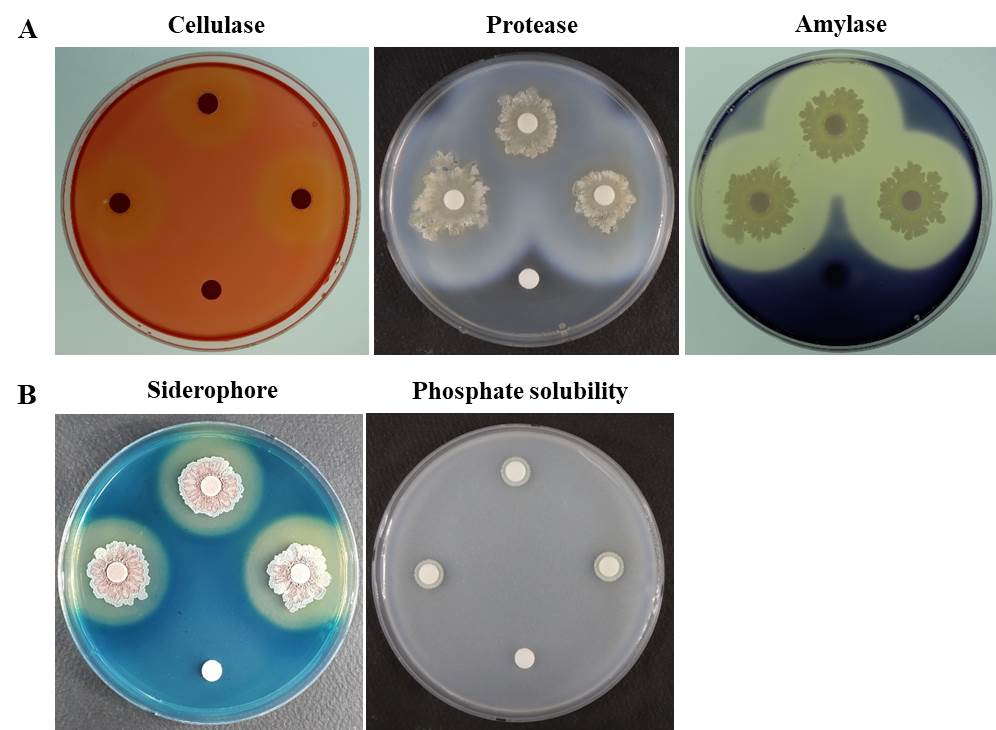


**Figure S3. Characterization of antagonistic substances produced by GYUN-300 strain.** (**A**) Detection of cellulase, protease, and amylase enzymes by *B. tequilensis* GYUN-300. (**B**) Siderophore production and inorganic phosphate solubilization assay *in vitro* by GYUN-300.


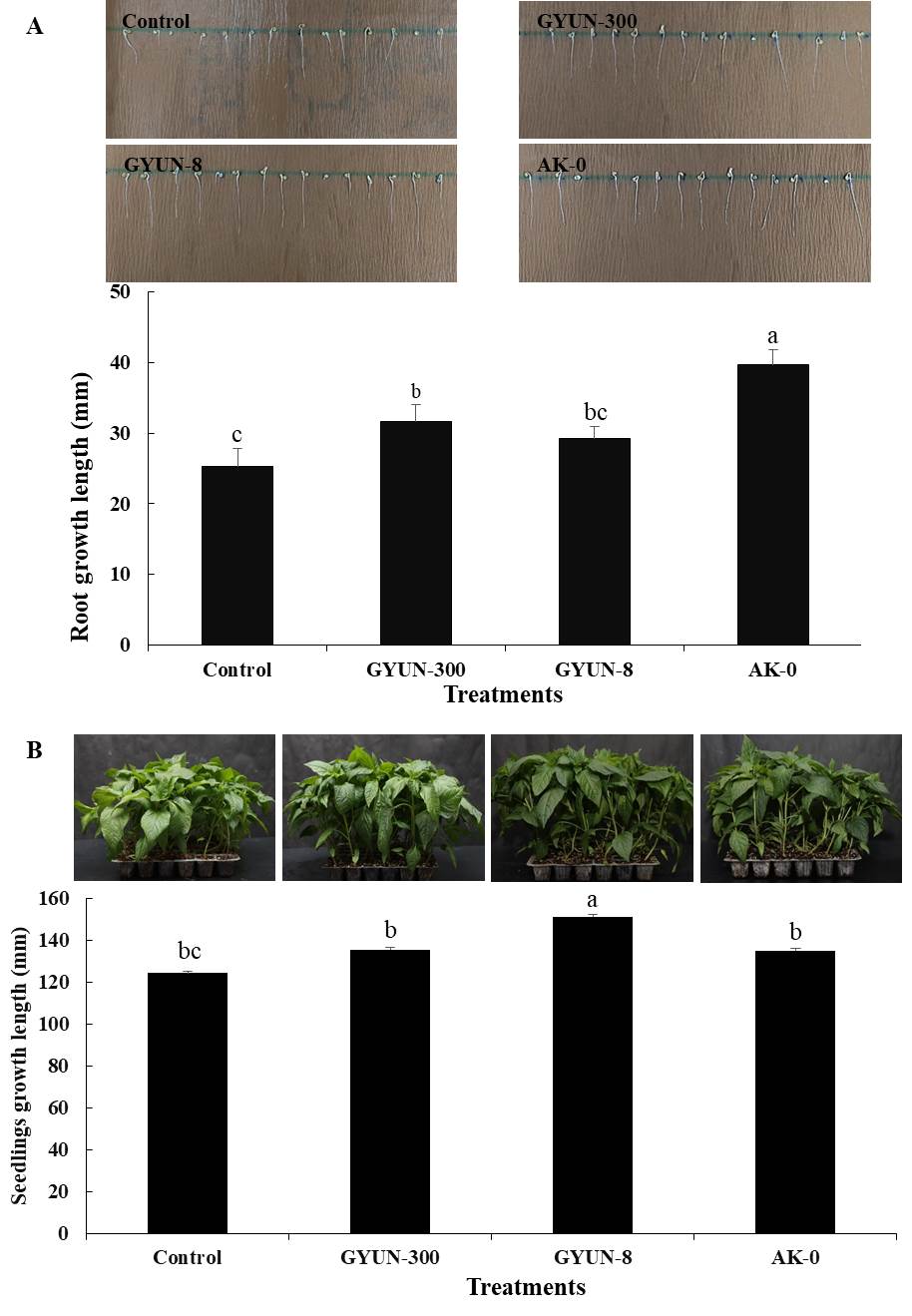


**Figure S4. Effect of GYUN-300 treatment on growth promotion of red pepper.** (**A**) Effect of treatment with GYUN-300, GYUN-8, or AK-0 bacterial suspensions (1 × 10^6^ CFU/mL) on seed germination of red pepper seedlings length (mm) in comparison with a non-treated control (water) under *in vitro* conditions using a double-layered wet paper method. (**B**) Effect of treatment with GYUN-300, GYUN-8, or AK-0 bacterial suspensions (1 × 10^6^ CFU/mL) on growth promotion of red pepper seedlings in comparison to non-treated control (water) under greenhouse conditions. The experiment was repeated at least once with 20 replicates (seedlings) per treatment. Bars with the same letters do not differ significantly between each other according to the least significant difference (LSD; *p* < 0.05).
